# Healthcare worker infected with Middle East Respiratory Syndrome during cardiopulmonary resuscitation in Korea, 2015

**DOI:** 10.4178/epih.e2017052

**Published:** 2017-11-12

**Authors:** Hae-Sung Nam, Mi-Yeon Yeon, Jung Wan Park, Jee-Young Hong, Ji Woong Son

**Affiliations:** 1 Department of Preventive Medicine and Public Health, Chungnam National University School of Medicine, Daejeon, Korea; 2 Division of Infectious Disease Surveillance, Korea Centers for Disease Control and Prevention, Cheongju, Korea; 3 Department of Preventive Medicine, Konyang University College of Medicine, Daejeon, Korea; 4 Department of Internal Medicine, Konyang University College of Medicine, Daejeon, Korea

**Keywords:** Middle East Respiratory Syndrome, Epidemiology, Infectious disease transmission, Patient-to-professional, Cardiopulmonary resuscitation, Korea

## Abstract

**OBJECTIVES:**

During the outbreak of the Middle East Respiratory Syndrome (MERS) in Korea in 2015, the Korea Centers for Disease Control and Prevention (KCDC) confirmed a case of MERS in a healthcare worker in Daejeon, South Korea. To verify the precise route of infection for the case, we conducted an in-depth epidemiological investigation in cooperation with the KCDC.

**METHODS:**

We reviewed the MERS outbreak investigation report of the KCDC, and interviewed the healthcare worker who had recovered from MERS. Using the media interview data, we reaffirmed and supplemented the nature of the exposure.

**RESULTS:**

The healthcare worker, a nurse, was infected while performing cardiopulmonary resuscitation (CPR) for a MERS patient in an isolation room. During the CPR which lasted for an hour, a large amount of body fluid was splashed. The nurse was presumed to have touched the mask to adjust its position during the CPR. She suggested that she was contaminated with the MERS patient’s body fluids by wiping away the sweat from her face during the CPR.

**CONCLUSIONS:**

The possible routes of infection may include the following: respiratory invasion of aerosols contaminated with MERS-coronavirus (MERS-CoV) through a gap between the face and mask; mucosal exposure to sweat contaminated with MERS-CoV; and contamination during doffing of personal protective equipment. The MERS guidelines should reflect this case to decrease the risk of infection during CPR.

## INTRODUCTION

The Middle East Respiratory Syndrome (MERS), which was first reported in Saudi Arabia in 2012, is caused by MERS-CoV, a human coronavirus (CoV). Similar to SARS-CoV, which causes severe acute respiratory syndrome (SARS), MERS-CoV can cause acute respiratory infections [1]. After the confirmation of the index case of MERS-CoV in South Korea (hereafter Korea) on May 20, 2015, its outbreak spread to 17 hospitals during May–July 2015 [2]. As one of the outbreak clusters, 25 nosocomial cases were reported in two hospitals located in Daejeon Metropolitan City, Korea [3]. Among the patients infected during the MERS outbreak, a healthcare worker was included, who was suspected to have been infected during cardiopulmonary resuscitation (CPR). The investigation of such a possible risk of exposure should provide useful information for improvement of infection prevention guidelines during CPR on patients with infectious respiratory diseases. The Private Epidemiological MERS Investigation Support Team of Daejeon Metropolitan City (hereafter the Daejeon in-depth investigation team) conducted an in-depth epidemiological investigation from June to September 2015. As a result of this investigation, we report a case of a healthcare worker who was infected by MERS-CoV during CPR in a general hospital located in Daejeon Metropolitan City, Korea.

## MATERIALS AND METHODS

Data sources for description of the case included the report of the Korea Centers for Disease Control and Prevention (KCDC) Investigation Team (hereafter the KCDC investigation report) from the KCDC, investigation data of the Daejeon in-depth investigation team, and public media data such as the reported interview contents [4,5] and the CPR scene [6]. The exposure situation and clinical characteristics of the infected case were described using the KCDC investigation report, and exposure situation was reconfirmed and supplemented based on investigation data of the Daejeon in-depth investigation team and public media data regarding the infected healthcare worker. Since this study was conducted as a MERS outbreak epidemiological investigation, it was not required to undergo review and approval processes of an institutional review board according to Article 2 of the Enforcement Decree of the Bioethics and Safety Act (Scope of Human Subject Study).

## RESULTS

### Overview of hospital outbreak

On May 28, 2015, the outbreak began when Case A, who was hospitalized in hospital A in Daejeon without diagnosing MERS infection, visited the emergency room of hospital B. That afternoon, Case A was admitted to a 6-bed capacity respiratory ward. Thereafter, 5 inpatients, 2 family caregivers and 1 professional caregiver were infected in the room where Case A stayed, and the MERS Case B was one of the inpatients. Case B who was an 82-yearold male admitted for bacterial pneumonia and asthma was exposed to Case A, and transferred to an isolated negative-pressure room in the surgical intensive care unit on May 30, 2015.

### Exposure situation of healthcare worker participating in cardiopulmonary resuscitation

On June 1, 2015, Case B experienced fever. On June 3, Case B had an aggravation of pneumonia leading to hypoxia and underwent CPR in the isolated room. The isolated negative-pressure room was designed to allow air to flow from the hallway to the window side. A large amount of hemoptysis was released during intubation, and came into contact with the bed sheet and personal protective equipment (PPE) of the healthcare workers. Hemoptysis was continuously observed while suctioning the airways. Although 6 healthcare workers wearing Level D PPE performed CPR for about 1 hour, Case B finally died, and was confirmed as a case of MERS on June 4, 2015.

Case C was a 39-year-old female nurse who participated in the above described CPR. In an interview with the Daejeon in-depth investigation team, she stated that she stayed in the isolated room for about 3 hours for CPR (1 hour) and to clean-up (2 hours), and that a large amount of body fluids splashed on her PPE during the CPR. According to a closed-circuit television (CCTV) video in the KCDC investigation team data, Case C was found to have stayed in the isolated room for about 3 hours, and touched the mask and goggle with her hands in contaminated gloves. In a press conference when returning to the hospital after complete recovery [4,5], Case C said, “I know that I was infected by MERS within body fluids of the patient when I unconsciously wiped off my sweat,” and also explained the situation of exposure during CPR, “Since the goggle was heavy, I thought it slid down together with the mask.”

### Clinical progress of Case C

Case C had no medical history that might have affected the onset of the infectious disease and its prognosis. On June 5, 2015, she experienced muscular pain at home after work, which intermittently appeared until June 10, 2015. On June 8, 2015, she experienced chills, but no fever. In the morning of June 11, 2015, Case C experienced abdominal pain while at work in the hospital; upon visiting the emergency room, her body temperature was 37.5°C. After being reported to the manager of the intensive care unit, Case C was isolated in a 1-bed ward in the afternoon of the same day. On June 11, 2015, a confirmatory test with a throat swab specimen yielded negative results, whereas a confirmatory test with sputum showed positive results on June 14, 2015. Case C was transferred to the national inpatient isolation units on the day of the confirmation. Finally, Case C completely recovered and was discharged on July 4, 2015.

### Quarantine of the contacted

Having been identified to have been in contact with Case C in the hospital, 169 people (51 patients and 118 hospital employees) were subjected to either cohort quarantine or home quarantine for 14 days. However, there were no more newly infected people.

## DISCUSSION

This case appears to be the first report of infection by MERS-CoV during CPR in the world including the Middle East. Although there were multiple cases of healthcare workers infected by MERS in hospitals, there was no case related with CPR [7,8]. In case of SARS that is similar to MERS, there was a case suspected to be infected during CPR. In 2003, Christian et al. [9] reported that a healthcare worker was infected during CPR for a patient with SARS.

Since Case C was found to have no contact with other MERS patients in the hospital except the corresponding CPR situation, Case C was suspected to have been infected during CPR for Case B. When judged based on non-specific symptoms such as muscular pain and chills, the incubation period of Case C seemed to be 2 days, while it could be 8 days if calculated based on fever. In infection of healthcare workers, manifestation of symptoms is likely to be delayed, because their basal health conditions are generally good. However, Case C showed non-specific symptoms relatively early, which suggests that Case C was exposed to a high level of MERS-CoV during CPR.

Despite the paucity of literature regarding the invasion route of MERS-CoV [10], potential infection routes of the present case can be speculated through the addition of literature for the invasion route of SARS [9,11], which are as follows (Table 1).

Respiratory invasion of aerosols contaminated with MERS-CoV (potential infectious route 1): It is possible that there was sweat during CPR. While the upper body was moving, the mask and goggle could lose close contact with the face, and then slide down, which could create a gap that air could flow in. It seemed that Case C touched the goggle and mask to try to reposition them. In this case, it is possible to be exposed to droplets or aerosols that were contaminated with MERS-CoV. CPR is an aerosol-generating procedure, so air-borne precautions are required [12]. During CPR, aerosols can be generated during intubation, suctioning of body fluids, chest compression, manual ventilation, and defibrillation [13].

Invasion by MERS-CoV-contaminated sweat through mucous membranes (potential infectious route 2): It is also possible that body fluids of the MERS patient came into contact with the face of Case C, passed underneath the goggle or the mask in a mix with sweat, and then invaded through mucous membranes of the eyes, nose or the mouth [11]. This route may include both cases in which body fluids of the CPR patient were splashed directly to the face of Case C and if contaminated gloves came into contact with the face.

Contamination with MERS-CoV while removing the PPE (potential infectious route 3): Contamination also frequently occurs while doffing the PPE [14,15]. Areas including the neck, foot, and head are frequently contaminated during doffing [15]. In the present case, it is also possible that Case C might have been contaminated with MERS-CoV whiles doffing.

The present case suggests the following implications for prevention of infection: First, it seemed that prolonged CPR mainly contributed to MERS infection in the present case. Considering the age, underlying disease and the large amount of continuous hemoptysis from the CPR subject, it was postulated that it exceeded the effective length of CPR time [16]. Family consent is an important consideration to stop CPR in the Korean medical culture. In the present case, it seemed that CPR was prolonged due to delayed family consent. To address this issue, the length of CPR time should be limited for patients with highly infectious diseases from the medical standpoint. Regarding ethics, it should be discussed for how long CPR should be performed, as the performer might stand the risk of infection.

Second, healthcare workers who perform CPR for MERS patients as in the present case should wear Level C PPE (composed of chemically resistant clothing, powered air-purifying respirator [PAPR], chemically resistant gloves, and chemically resistant boots) rather than Level D PPE (composed of whole-body gowns, N95 equivalent mask, gloves, goggles or face shield, and shoe covers) [17]. During CPR for a MERS patient, it is possible that aerosols contaminated with MERS-CoV could be generated and excretion from the patient could be splashed on the performer’s face. When wearing Level D PPE, the CPR performer may experience short-breath and sweat due to physical exertion. If the mask and goggle are not tightly adhered to the face, a gap may become a route of MERS-CoV infection. Level C PPE should be able to address most of such issues. If Level D PPE is used in the absence of Level C PPE, PAPR with a hood should be added instead of a mask and goggle. While these were not included in the 2015 KCDC guidelines for management of MERS [18], most of them were included in the 2016 guidelines [19]. In the future MERS countermeasure revision process, preparation and application of Level C PPE for emergency situations such as CPR should be discussed.

Since this in-depth epidemiological investigation failed to obtain the CCTV video, it was unable to secure objective evidence for a detailed situation of the exposure. However, information related to potential exposure routes were identified through interview and press report data. These data sources were useful for elaborating exposure situation that was described in the initial survey data, the KCDC investigation report.

In summary, the present case involved the infection of a healthcare worker by MERS-CoV during CPR, in which identified potential infection routes included respiratory invasion through aerosols, exposure of mucous membrane to contaminated sweat, and contamination during doffing. This case suggests the need to revise the guidelines for management of MERS in terms of performing CPR and wearing PPE.

## Figures and Tables

**Table 1.** The possible routes of infection of the healthcare worker with MERS-CoV during CPR and control measures including PPE

Route of infection	Possible mechanism	Refuting evidence	Control measures	PPE for prevention
Respiratory invasion of aerosols contaminated with MERS-CoV during CPR	CPR is an aerosol-generating procedure	The CPR was performed in a negative pressured room	Standard, contact, and airborne precautions	Level C is preferred to Level D for airborne precaution; PAPR should be added when using Level D equipment
	Failure of mask or goggles in seal- ing tightly to the wearer’s face during 1 hr CPR			
Mucosal exposure to sweat contaminated with MERS-CoV during CPR	The face of healthcare worker contaminated with the splashed body fluid from infector	-	Standard and contact precautions	Level C is preferred to Level D to protect from the body fluid of MERS patient
	Intrusion of the contaminated sweat through gaps between face and mask/goggles			
Contamination of body with MERS-CoV during doffing of PPE after CPR	Contaminated body or hand dur- ing doffing of PPE	The healthcare worker performed hand hygiene immediately after CPR	Standard and contact precautions	-

MERS-CoV, Middle East Respiratory Syndrome-coronavirus; CPR, cardiopulmonary resuscitation; PAPR, powered air-purifying respirator; PPE, personal protective equipment.
