# Supplementary material for: Healthcare worker infected with Middle East Respiratory Syndrome during cardiopulmonary resuscitation in Korea, 2015
Source: Epidemiol Health. 2017 Nov 12;39:e2017052. doi: 10.4178/epih.e2017052 (PMC5733382; doi:10.4178/epih.e2017052)
Supplement: Supplementary file 1 [file epih-39-e2017052-supplementary.pdf]

Volume: 39, Article ID: e2017052
https://doi.org/10.4178/epih.e2017052

Healthcare worker infected with Middle East respiratory syndrome during cardiopulmonary resuscitation in Korea, 2015

심폐소생술 시행 중 MERS-CoV에 감염된 의료인 사례

남해성[1], 연미연[1], 박정완[2], 홍지영[3], 손지웅[4]

[1]충남대학교 의과대학 예방의학교실, 대전; [2]질병관리본부, 청주; [3]건양대학교 의과대학 예방의학교실, 대전; [4]건양대학교 의과대학 호흡기내과학교실, 대전

Hae-Sung Nam[1], Mi-Yeon Yeon[1], Jung Wan Park[2], Jee-Young Hong[3], Ji Woong Son[4]

[1]Department of Preventive Medicine and Public Health, Chungnam National University School of Medicine, Daejeon; [2]Division of Infectious Disease Surveillance, Korea Centers for Disease Control and Prevention, Cheongju; [3]Department of Preventive Medicine, College of Medicine, Konyang University, Daejeon; [4]Department of Internal Medicine, College of Medicine, Konyang University, Daejeon

Corresponding Author: Hae-Sung Nam

Dept. of Preventive Medicine and Public Health, Chungnam National University School of Medicine, 266 Munhwa-ro, Daejeon, 35015, Korea
E-mail:hsnam@cnu.ac.kr
Tel)+82-42-580-8266
Fax)+82-42-583-7561

Running title: MERS infection during CPR

Volume: 39, Article ID: e2017052
https://doi.org/10.4178/epih.e2017052

<ABSTRACT>

OBJECTIVES

During the Korean outbreak of Middle East respiratory syndrome (MERS) in 2015, Korea Centers for Disease Control and Prevention (KCDC) confirmed the MERS case of healthcare worker in Daejeon, South Korea. To verify the precise route of infection for the case, we conducted an in-depth epidemiological investigation in cooperation with KCDC.

METHODS

We reviewed the MERS outbreak investigation report of KCDC, and conducted an interview with the healthcare worker who was recovered from MERS. Using the media interview data, we reaffirmed and supplemented the situation of exposure.

RESULTS

The healthcare worker, a nurse, was infected when performing cardiopulmonary resuscitation (CPR) for a MERS patient in an isolation room. During the CPR for an hour, a large amount of fluid was splashed. The nurse was presumed to have touched the mask to adjust its position during the CPR. She suggested that she was contaminated with the MERS patient's body fluids by wiping away the sweat on her face during the CPR.

CONCLUSIONS

The estimated routes of infection may include as follows: (1) respiratory invasion of aerosols contaminated with MERS-CoV through a gap between face and mask; (2) mucosal exposure to sweat contaminated with MERS-CoV; (3) contamination during doffing of personal protective equipment. The MERS guidelines should reflect this case to decrease the risk of infection during CPR.

Keywords: Middle East Respiratory Syndrome, Epidemiology, Infectious disease transmission, Patient-to-professional, Cardiopulmonary resuscitation, Korea

Volume: 39, Article ID: e2017052
https://doi.org/10.4178/epih.e2017052

서론 (Introduction)

2012년 사우디아라비아에서 처음 보고된 중동호흡기증후군(Middle East respiratory syndrome, MERS)의 원인균은 인간 coronavirus(CoV)의 변형인 MERS-CoV이다. MERS-CoV는 중증급성호흡기증후군(the Severe Acute Respiratory Syndrome, SARS)을 일으키는 SARS-CoV와 유사하게 급성 호흡기 감염증상을 일으킬 수 있다[1]. 2015년 5월 20일 한국에서 첫 Middle East respiratory syndrome-coronavirus (MERS-CoV) 환자가 확진된 후, 2015년 5~7월 기간 전국 17개 병원으로 유행이 확산되었다[2]. 이 유행 군집의 하나로서 대전광역시에 소재한 A병원과 B병원에서도 25명의 원내 감염 환자가 발생하였다[3]. 감염 사례 중에는 MERS 환자 심폐소생술(cardio–pulmonary resuscitation, CPR) 도중 전염된 것으로 추정되는 의료인 사례도 있었다. 이러한 사례에서 감염 폭로 상황을 파악해 보는 것은 향후 심각한 호흡기 감염병 환자의 CPR 수행 시 감염 예방 수칙을 개선하는데 유용한 정보를 줄 수 있을 것이다. 대전광역시 메르스 대응 민간역학조사지원단(이하 민간역학조사지원단)에서는 2015년 6월부터 9월까지 기간 동안 심층역학조사 활동을 실시한 바 있다. 이 조사결과의 하나로 대전광역시의 한 종합병원에서 심폐소생술 시행 중 MERS-Cov에 감염된 의료인 사례를 보고하고자 한다.

방법 (Methods)

사례 기술을 위한 자료원으로 질병관리본부(the Korea Centers for Disease Control and Prevention)의 중앙역학조사반(the KCDC investigation team)의 메르스 확진자 역학조사 결과보고 자료(이하 중앙역학조사반 자료), 민간역학조사지원단(the Daejeon in-depth investigation team)의 조사 자료, 대중매체에 보도된 인터뷰 내용[4-5]과 심폐소생술 장면[6]

등을 이용하였다. 중앙역학조사반 자료를 이용하여 감염자의 폭로 상황과 임상적 특성을 기술하였고, 민간역학조사지원단의 대상자 면담 자료와 대중매체 자료를 통해 폭로 상황을 재확인 및 보완하였다. 본 연구는 메르스 유행 역학조사로서 수행되었기 때문에 생명윤리 및 안전에 관한 법률 시행규칙 제2조(인간대상연구의 범위)에 의거하여 별도의 기관윤리위원회 심사 및 승인절차는 필요하지 않았다.

결과 (Results)

병원유행의 개요

2015년 5월 28일 대전의 A병원에 MERS 감염을 인지하지 못한 상태에서 입원해 있던 사례 A가 퇴원 후 B병원 응급실에 방문하면서 병원 내 유행이 시작되었다. 당일 오후 사례 A는 호흡기병동의 6인실에 입원하였다. 이후 사례 A가 입원했던 병실에서는 입원자 5명, 가족 간병인 2명, 직업간병인 1명이 감염되었는데 MERS 사례 B도 그 입원자 중 한 명이었다. 82세 남성으로 세균성 폐렴과 천식으로 입원 중이던 사례 B는 사례 A에 폭로된 후, 5월 30일 격리조치 위해 외과계 중환자실 구역에 위치한 음압 격리실로 전실되었다.

심폐소생술 참여 의료진의 폭로 상황

6월 1일 사례 B는 발열 증상을 보였으며, 6월 3일 폐렴이 악화되어 저산소증이 나타나자 입원해 있던 격리실에서 곧바로 CPR을 받았다. 음압실의 공기 흐름은 복도에서 창문쪽을 향하도록 설계되어 있었다. 기관삽관(intubation) 과정에서 다량의 객혈이 배출되었고, 침상 시트와 의료진의 방호복에 묻었다. 기도 체액 제거 시 객혈이 지속적으로 관찰

되었다. D등급 방호복(Level D protector)을 입은 의료진 6명이 CPR을 약 1시간 가량 시행하였으나, 사례 B는 결국 사망하였고 6월 4일 MERS로 확진되었다.

사례 C는 상기 CPR에 참여하였던 39세 여성 간호사였다. 민간역학조사지원단과의 면담에서 당시 CPR 수행(1시간)과 정리(2시간)을 위해 3시간 동안 격리실에 머물렀고, CPR 수행 중에 다량의 체액이 튀어서 보호복에 묻었다고 진술하였다. 중앙역학조사반 자료에 의하면 Closed-circuit television (CCTV) 관찰에서 약 3시간 동안 격리실에 머물렀고, 오염된 장갑을 낀 손으로 마스크와 고글을 만졌던 것으로 파악되었다. 사례 C는 완치 후 병원복귀 기자회견[4-5]에서 "무의식 중에 흐르는 땀을 닦다가 환자의 체액에 노출되어 메르스에 감염된 것으로 알고 있습니다"라고 밝혔고, "고글이 무거우니까 마스크랑 같이 흘러내리는 생각은 들었거든요"라고 CPR 당시 폭로 상황에 대해 설명하였다.

사례 C의 임상 경과

감염병 발생과 예후에 영향을 줄 수 있는 질병 과거력은 없었다. 6월 5일 저녁 퇴근 후 근육통을 느꼈고 이후 6월 10일까지 간헐적으로 나타났다. 6월 8일 오한이 있었으나 발열은 없었다. 6월 11일 오전 병원 근무 중 복통이 있어 응급실 방문하였는데, 당시 체온은 37.5°C였다. 중환자실 관리자에게 이를 보고하여 당일 오후에 1인실에 격리되었다. 6월 11일 인후도말 검체에 대한 확진 검사에서는 음성이었으나, 6월 14일 객담에 대한 확진 검사에서 양성으로 판정되었다. 이에 따라 확진 당일 국가지정 격리병상 시설로 전원하였고, 7월 4일 완치 퇴원하였다.

접촉자 검역

Volume: 39, Article ID: e2017052
https://doi.org/10.4178/epih.e2017052

사례 C와 병원에서 밀접 접촉한 것으로 파악되어 14일간 코호트 검역 또는 자가 검역 조치되었던 대상자는 169명(환자 51명, 병원 직원 118명)이었는데 이들 중 추가감염자는 없었다.

Volume: 39, Article ID: e2017052
https://doi.org/10.4178/epih.e2017052

Table 1. The possible routes of infection of the health worker with MERS-CoV during cardiopulmonary resuscitation and control measures including personal protective equipment

Route of infection	Possible mechanism	Refuting evidence	Control measures	PPE for prevention
Respiratory invasion of aerosols contaminated with MERS-CoV during CPR	CPR is an aerosol-generating procedure; Failure of mask or goggles in sealing tightly to the wearer's face during 1 hr CPR	The CPR was performed in a negative pressured room	Standard, contact, and airborne precautions	Level C is preferred to level D for airborne precaution; PAPR should be added when using level D equipment
Mucosal exposure to sweat contaminated with MERS-CoV during CPR	The face of health worker contaminated with the splashed body fluid from infector; Intrusion of the contaminated sweat through gaps between face and mask/goggles	-	Standard and contact precautions	Level C is preferred to level D to protect from the body fluid of MERS patient
Contamination of body with MERS-CoV during doffing of PPE after CPR	Contaminated body or hand during doffing of PPE	The health worker performed hand hygiene immediately after CPR	Standard and contact precautions	-

MERS-CoV, Middle East respiratory syndrome-coronavirus; PAPR, Powered air-purifying respirator; PPE, personal protective equipment

Volume: 39, Article ID: e2017052
https://doi.org/10.4178/epih.e2017052

토의 (Discussion)

CPR 도중 MERS-CoV에 감염된 본 사례는 중동지역을 포함하여 전세계적으로 볼 때 처음 보고되는 것으로 보인다. 병원환경에서 MERS에 감염된 의료인 사례는 다수 찾을 수 있으나 CPR과 관련된 사례는 없었다[7, 8]. MERS와 유사한 SARS의 경우에는 CPR 도중 감염된 것으로 추정되는 사례가 있었는데, Christian et al.[9]은 2003년 SARS 환자의 CPR 도중 의료인이 감염된 사례를 처음 보고한 바 있다.

사례 C가 해당 CPR 이외에 원내에서 다른 MERS 환자와 접촉했던 상황은 없었던 것으로 파악되었기 때문에, 사례 C는 사례 B에 대한 CPR 수행 중 감염된 것으로 보인다. 사례 C의 잠복기는 근육통, 오한 등 비특이적 증상을 기준으로 할 때 2일, 발열을 기준으로 할 때 8일로 추정된다. 의료인 감염 사례들은 일반적으로 감염 전 건강수준이 양호한 상태여서 비교적 증상이 늦게 나타나는데, 사례 C는 비특이적 증상이 비교적 일찍 나타났다. 이는 CPR과정에서 MERS-CoV에 고농도로 폭로되었을 가능성을 시사한다.

MERS-CoV의 침입경로에 대한 문헌이 부족하기는 하지만[10], SARS의 침입경로에 관한 문헌을 추가하여[9, 11] 본 사례에서 가능한 감염경로를 추정해보면 다음과 같다(Table 1).

- MERS-CoV에 오염된 에어로졸의 호흡기 침투 가능성 (추정 감염경로 1): CPR 수행 동안 땀이 흐르고 상체의 움직임이 있는 상황에서 마스크와 고글이 안면부에 밀착되지 못하고 흘러내려 실내 공기가 들어올 틈이 발생했을 수 있다. 사례 C가 고글과 마스크를 만진 것도 이를 복구하려는 시도로 보인다. 이러한 경우 MERS-CoV에 오염된 비말이나 에어로졸에 노출될 수 있다. CPR은 에어로졸 발생가능성

이 높은 시술(aerosol-generating procedure)로 공기매개 전파에 대한 주의(air-borne precautions)가 필요하다[12]. CPR에서는 기도 삽관(intubation), 기도 체액 제거(suction of body fluids), 흉부압박(chest compression), 수동환기(manual ventilation), 제세동(defibrillation) 과정 중에 에어로졸 발생이 가능하다[13].

- MERS-CoV에 오염된 땀의 점막 침투 가능성 (추정 감염경로 2): 또 다른 가능성으로 MERS 환자의 체액이 사례 C의 안면부에 묻으면서 땀과 함께 섞여 고글 또는 마스크 안으로 스며들어 눈, 코, 입 등 안면부의 점막을 통해 MERS-CoV가 침투했을 수도 있다[11]. 이 경로는 CPR 대상자의 체액이 사례 C의 안면부에 직접 튀는 경우와 오염된 장갑으로 안면부를 접촉한 경우 모두 가능하다.
- 보호구 탈의 과정에서 MERS-Cov에 오염 가능성 (추정 감염경로 3): 보호구 탈의 과정에서 오염 또한 자주 발생하는 문제이다[14-15]. 탈의과정에서 오염 빈도가 높은 부위로 목(neck), 발(foot), 머리(head) 등이 보고된 바 있다[15]. 본 사례도 탈의 과정 중에 MERS-Cov에 오염되었을 가능성이 있다.

본 사례를 참고하여 향후 감염예방을 위한 시사점을 제시하면 다음과 같다. 첫째, 본 사례의 경우 지나치게 긴 CPR 수행시간이 MERS 감염의 주요 기여 요인으로 보인다. CPR 대상자의 연령, 기저질환, 다량의 지속적인 객혈 등을 감안하면 유효한 CPR 수행시간을 초과한 것으로 추정된다[16]. 한국의 의료문화에서 CPR을 중단하기 위해서는 가족의 동의가 중요한 고려사항이 된다. 본 사례에서는 가족의 동의가 지연되어 CPR 수행시간이 길었던 것으로 보인다. 이러한 문제를 해결하기 위해서는 중증도가 높은 감염질환의 경우 CPR 수행시간을 의학적 관점에서 제한하는 지침이 필요하다고 본다. 아울러, CPR

Volume: 39, Article ID: e2017052
https://doi.org/10.4178/epih.e2017052

수행자가 감염 위험에 처할 수 있는 상황에서 CPR 처치를 얼마 동안 지속해야 하는가에 대한 윤리적 측면의 논의도 필요할 것이다.

둘째, 본 사례와 같이 MERS 환자의 CPR 시에는 의료진이 D등급 보호구(전신보호복, N95 동급의 마스크, 장갑, 보안경(goggles) 또는 안면보호구(face shield), 신발덮개 등으로 구성) 대신 C등급 보호구(내화학 보호복, 전동식 공기정화 호흡기보호구(powered air-purifying respirator, PAPR), 내화학 장갑, 내화학 장화 등으로 구성)를 사용하도록 권고되어야 할 것으로 생각된다[17]. MERS 환자의 CPR 시에는 MERS-CoV로 오염된 에어로졸이 생성될 수 있고 환자의 분비물이 CPR 수행자의 얼굴에 튈 수도 있다. D등급 보호구를 착용한 경우 CPR 수행자는 활발한 신체활동으로 호흡이 가쁘고 땀이 날 수 있는데, 이때 마스크와 보안경이 얼굴에 밀착되지 못하여 MERS-CoV가 유입되는 틈이 발생할 수 있다. C등급 보호구를 사용한다면 이러한 문제를 대부분 해결할 수 있을 것이다. 만약 C등급 보호구가 없어 D등급 보호구를 착용해야 하는 경우라면 마스크와 보안경 대신 후드가 있는 PAPR이 추가되어야 할 것이다. 2015년 질병관리본부의 메르스 대응 지침[18]에서는 이러한 사항이 포함되지 않았지만 2016년 지침[19]부터는 대부분 포함되었다. 향후 메르스 대응 지침 개정과정에서는 CPR과 같은 응급상황에 대비하여 C등급 보호구의 구비와 사용에 대한 논의가 필요할 것으로 사료된다.

본 심층역학조사에서는 CCTV 녹화물을 획득하지 못하여 폭로의 세부 상황에 대한 객관적 증거를 확보할 수는 없었다. 하지만 면담과 언론보도 자료를 통해 가능성 있는 폭로 경로에 관한 정보를 파악할 수 있었다. 이들 자료원은 초기 역학조사 자료(중앙역학조사반 자료)에서 기술된 폭로 상황을 구체화 하는 데 유용하였다.

요약하면, 본 사례는 CPR 중 의료진이 MERS-CoV에 감염된 경우로 에어로졸을 통한 호흡기 침투, 오염된 땀에 점막 노출, 탈의 과정에서 오염 등이 가능한 감염 경로로 파악되었다. 본 사례는 MERS 환자에 대한 CPR 수행 및 수행자의 보호구 착용과 관련하여 MERS 관리 지침의 개정이 필요함을 시사한다.

감사의 글 (Acknowledgements)

This work was supported by research fund of Chungnam National University.

참고문헌 (References)

1. Raj VS, Osterhaus AD, Fouchier RA, Haagmans BL. MERS: emergence of a novel human coronavirus. Curr Opin Virol 2014;5:58-62.
2. Ki M. 2015 MERS outbreak in Korea: hospital-to-hospital transmission. Epidemiol Health 2015;37:e2015033.
3. Nam HS, Park JW, Ki M, Yeon MY, Kim J, Kim SW. High fatality rates and associated factors in two hospital outbreaks of MERS in Daejeon, the Republic of Korea. Int J Infect Dis 2017;58:37-42.
4. Doctors News. " I was fine to be sick, but I was afraid to infect others"; 2015 Jul 7 [cited 2015 Dec 28]. Available from: http://www.doctorsnews.co.kr/news/articleView.html?idxno=104719 (Korean).
5. Daejeon MBC News. "I'll always be with patients"; 2015. Jul 7 [cited 2015 Dec 28]. Available from: https://www.youtube.com/watch?v=HG0O4MfwHLY (Korean).
6. YTN. " Thank you very much for the support of the people" 2015. Jul 7 [cited 2015 Dec 28]. Available from: https://www.youtube.com/watch?v=690yZPOrWCw (Korean).
7. Maltezou HC, Tsiodras S. Middle East respiratory syndrome coronavirus: implications for health care facilities. Am J Infect Control 2014;42:1261-5.
8. Hunter JC, Nguyen D, Aden B, Al Bandar Z, Al Dhaheri W, Abu Elkheir K, Khudair A, Al Mulla M, El Saleh F, Imambaccus H, Al Kaabi N, Sheikh FA, Sasse J, Turner A, Abdel Wareth L, Weber S, Al Ameri A, Abu Amer W, Alami NN, Bunga S, Haynes LM, Hall AJ, Kallen AJ, Kuhar D, Pham H, Pringle K, Tong S, Whitaker BL, Gerber SI, Al Hosani FI. Transmission of Middle East Respiratory Syndrome Coronavirus Infections in Healthcare

Settings, Abu Dhabi. Emerg Infect Dis 2016;22:647-56.
9. Christian MD, Loutfy M, McDonald LC, Martinez KF, Ofner M, Wong T, Wallington T, Gold WL, Mederski B, Green K, Low DE. Possible SARS coronavirus transmission during cardiopulmonary resuscitation. Emerg Infect Dis 2004;10:287-93.
10. Zumla A, Hui DS, Perlman S. Middle East respiratory syndrome. Lancet 2015;386:995-1007.
11. Otter JA, Donskey C, Yezli S, Douthwaite S, Goldenberg SD, Weber DJ. Transmission of SARS and MERS coronaviruses and influenza virus in healthcare settings: the possible role of dry surface contamination. J Hosp Infect 2016;92:235-50.
12. Zumla A, Hui DS. Infection control and MERS-CoV in health-care workers. Lancet 2014;383:1869-71.
13. Tran K, Cimon K, Severn M, Pessoa-Silva CL, Conly J. Aerosol generating procedures and risk of transmission of acute respiratory infections to healthcare workers: a systematic review. PLoS One 2012;7:e35797.
14. Kwon JH, Burnham CD, Reske KA, Liang SY, Hink T, Wallace MA, Shupe A, Seiler S, Cass C, Fraser VJ, Dubberke ER. Assessment of Healthcare Worker Protocol Deviations and Self-Contamination During Personal Protective Equipment Donning and Doffing. Infect Control Hosp Epidemiol 2017:1-7.
15. Lim SM, Cha WC, Chae MK, Jo IJ. Contamination during doffing of personal protective equipment by healthcare providers. Clin Exp Emerg Med 2015;2:162-7.
16. Lee SW, Han KS, Park JS, Lee JS, Kim SJ. Prognostic indicators of survival and survival prediction model following extracorporeal cardiopulmonary resuscitation in patients with sudden refractory cardiac arrest. Ann Intensive Care 2017;7:87.
17. Kim JY, Song JY, Yoon YK, Choi SH, Song YG, Kim SR, Son HJ, Jeong SY, Choi JH, Kim KM, Yoon HJ, Choi JY, Kim TH, Choi YH, Kim HB, Yoon JH, Lee J, Eom JS, Lee SO, Oh WS, Yoo JH, Kim WJ, Cheong HJ. Middle East Respiratory Syndrome Infection Control and Prevention Guideline for Healthcare Facilities. Infect Chemother 2015;47:278-302.
18. Korea Centers for Disease Control and Prevention (KCDC). Guideline for management of MERS. Cheongju: Korea Centers for Disease Control and Prevention; 2015 June, p. 50 (Korean).
19. Korea Centers for Disease Control and Prevention (KCDC). Guideline for management of MERS. Cheongju: Korea Centers for Disease Control and Prevention; 2016 July, p. 161 (Korean).
